# Acute caffeine supplementation and resistance exercise performance in female participants: a systematic review and three-level meta-analysis

**DOI:** 10.3389/fphys.2026.1892370

**Published:** 2026-07-29

**Authors:** Li Liang, Ming Chen, Bo Qi

**Affiliations:** 1 Department of Physical Education, Central South University, Changsha, China; 2 Institute of Physical Education, Kunming University of Science and Technology, Kunming, China

**Keywords:** caffeine, crossover trials, female athletes, female-specific evidence, muscular endurance, muscular strength, resistance exercise, three-level meta-analysis

## Abstract

**Background and objectives:**

Caffeine is a widely studied ergogenic aid, but evidence for its acute effects on resistance exercise performance is primarily derived from male or mixed-sex samples, with scarce data specific to women. Previous women-specific syntheses did not model the multiple dependent outcomes extracted from the same crossover trial, which may have produced overly narrow uncertainty estimates.

**Methods:**

PubMed, Web of Science Core Collection, Scopus and Cochrane Library were systematically searched through 26 April 2026 to include randomized, placebo-controlled trials testing acute, dose-defined caffeine before resistance exercise in female participants or independently extractable female subgroups. Effect sizes were paired Hedges’ g and were synthesized separately for six outcome domains using three-level random-effects models with cluster-robust variance estimation: maximal strength, muscular endurance, movement speed, functional strength, functional endurance, and total load. 95% confidence intervals and prediction intervals were reported, and RoB 2 and GRADE were used to assess risk of bias and certainty of evidence.

**Results:**

A total of 24 studies were included (23 of which were included in the quantitative synthesis, with 107 effect sizes). Maximal strength showed a small mean effect (g = 0.17, 95% CI 0.06 to 0.29; low GRADE); muscular endurance showed a larger mean effect (g = 0.54, 95% CI 0.06 to 1.02; low GRADE), but its wide prediction interval and influence diagnostics indicated low reproducibility across protocols; movement speed showed a small, model-based estimate, but cluster-robust inference was uncertain because only three study clusters were available (g = 0.13; CR2 p = 0.072; very low GRADE). The study clusters for functional strength, functional endurance, and total load were too small and were not pooled for inference, so these domains are summarized descriptively. Meta-regressions of caffeine dosage, mean age of participants, and resistance load did not identify reliable moderation.

**Conclusion:**

Acute caffeine may slightly improve maximal strength and may benefit muscular endurance in some protocols, but the certainty of evidence is low or very low for most outcomes and prediction intervals indicate limited reproducibility. Current evidence therefore does not support strong recommendations by dosage, age, resistance load, menstrual cycle phase, hormonal status, or habitual caffeine intake. Future trials should report menstrual cycle phase, hormonal contraceptive use, menopausal status, habitual caffeine intake, adverse symptoms, sleep disturbance, blinding integrity, washout duration, and paired/change-score data with within-participant correlations.

**Systematic review registration:**

https://osf.io/2msf6/overview, identifier 10.17605/OSF.IO/2MSF6.

## Graphical Abstract

**Figure:**
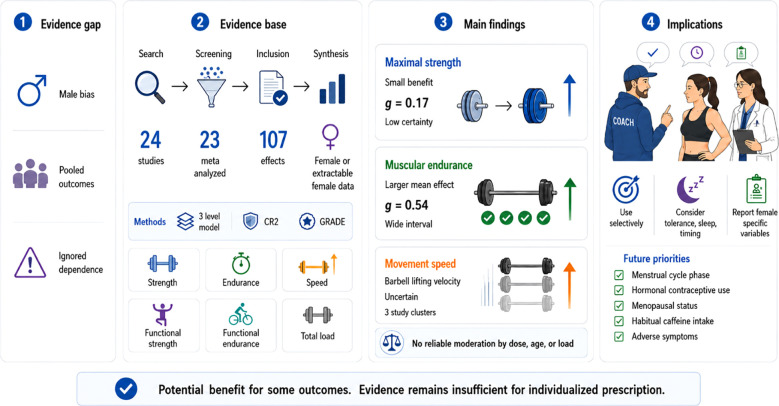

## 1 Introduction

Caffeine (1,3,7-trimethylxanthine) is among the most studied ergogenic aids in sports nutrition. Its primary mechanism is thought to involve antagonism of adenosine A1 and A2A receptors, increasing central nervous system excitability and reducing perceived exertion and pain during exercise (Fredholm et al., 1999; Davis et al., 2003; Doherty and Smith, 2005; Kalmar, 2005; Davis and Green, 2009). Peripheral effects on excitation-contraction coupling have also been proposed, although their contribution during whole-body resistance exercise is less certain. The International Society for Sports Nutrition (ISSN) position statement indicates that acute intake of caffeine at 3 to 6 mg·kg^-^¹ body mass can improve performance in multiple sports. Higher doses do not appear necessary for most ergogenic effects and may increase adverse effects such as gastrointestinal discomfort, anxiety, palpitations, and sleep disturbance (Guest et al., 2021). The International Olympic Committee consensus also lists caffeine as a well-documented performance-enhancing supplement, emphasizing that its use must be combined with sport characteristics, training goals, timing of intake, and individual tolerance (Maughan et al., 2018). In resistance exercise, acute caffeine intake can slightly improve maximal strength, muscular endurance, and movement speed, but these effects appear to be context-dependent and may vary by exercise pattern, load intensity, training level, and test task (Warren et al., 2010; Grgic et al., 2018; Southward et al., 2018; Astorino and Roberson, 2010). Evaluating the effect of caffeine on resistance exercise performance, therefore, requires distinguishing between outcomes such as maximal strength, number of repetitions to exhaustion, movement speed, total load, and functional performance; these outcomes reflect different task demands and should not be collapsed into a single performance construct without justification (Raya-González et al., 2020; Warren et al., 2010).

Although caffeine has been studied extensively in resistance exercise contexts, the evidence base is dominated by male or mixed-sex samples, with fewer trials designed to estimate effects in women. Because few caffeine and resistance exercise trials were designed to estimate effects in women, mixed-sample estimates provide limited information about female-specific uncertainty (Costello et al., 2014; Cowley et al., 2021). Bibliometric evidence shows that the proportion of female participants in sports medicine research is substantially lower than that of male participants, making it difficult to extrapolate overall conclusions to women (Costello et al., 2014). Insufficient female samples and the lack of sex-stratified analysis remain common in recent studies, and the average effect of mixed-sex samples can mask the effect characteristics and uncertainties specific to women (Cowley et al., 2021). Several pharmacokinetic, endocrine, genetic, and neuromuscular lines of evidence suggest that the caffeine response in women may show biologically plausible inter-individual heterogeneity: caffeine clearance via hepatic CYP1A2 may be slower in women than in men, and estrogen-containing oral contraceptives can further reduce clearance (Abernethy and Todd, 1985; Granfors et al., 2005; Grzegorzewski et al., 2022; Pickering and Kiely, 2018; Pollock et al., 1999); CYP1A2 and ADORA2A polymorphisms may contribute to variability in caffeine metabolism, adenosine-receptor signaling, perceived anxiety, sleep disturbance, and ergogenic response; and hormonal status may plausibly influence caffeine response across the menstrual cycle, although the net effect of these cyclical changes on motor performance appears small and the evidence is limited (Lara et al., 2014; McNulty et al., 2020; Smith, 2002). On average, women may also show greater fatigue resistance during some sustained submaximal contractions, although this varies by task, muscle group, contraction type, and training status (Hunter, 2014; Mielgo-Ayuso et al., 2019). These factors do not establish a different ergogenic response in women, but they make male-dominated or mixed-sex estimates an insufficient basis for female-specific recommendations and provide a methodological rationale for examining maximal strength and muscular endurance separately, rather than assuming a uniform caffeine response across outcomes (Elliott-Sale et al., 2021).

Existing studies have explored the effect of caffeine on women’s resistance exercise performance, but they are generally limited by small sample sizes, heterogeneous outcome definitions, incomplete paired data, and inconsistent reporting of menstrual and hormonal status (Grgic and Del Coso, 2021). The previous women-specific meta-analysis included eight crossover placebo-controlled trials and reported only a small positive effect of caffeine on muscle strength (SMD = 0.18) and muscular endurance (SMD = 0.25) in women, with subgroup effects on lower-limb strength and endurance that were not statistically significant; these conclusions lack stability and are sensitive to differences in sample size, test sites, exercise tasks, and design (Grgic and Del Coso, 2021). From a statistical perspective, trials of caffeine and resistance exercise in women often employ within-participant crossover designs, in which a single sample can contribute multiple dependent effect sizes across doses, time points, exercise tasks, and outcome measures. This produces highly correlated effect sizes within a study, violating the core assumption of traditional meta-analysis that effect sizes are independent (Hedges et al., 2010; Tipton, 2015; Pustejovsky and Tipton, 2022). Treating dependent effect sizes as independent can underestimate standard errors, inflate effective sample sizes, and excessively narrow confidence intervals, distorting the pooled results (Hedges et al., 2010; Tipton, 2015). At the same time, the various outcomes of resistance exercise are not interchangeable. Maximal strength, number of repetitions to exhaustion, movement speed, functional tests, and total load reflect different neuromuscular and metabolic demands, and pooling these outcomes into one estimate would obscure differences among maximal strength, muscular endurance, movement speed, and total load (Grgic et al., 2018; Raya-González et al., 2020; Warren et al., 2010). In recent years, several trials, conducted either exclusively in women or with extractable female data, have increased the number of eligible women-only or women-extractable datasets (Harty et al., 2020; Filip-Stachnik et al., 2022; Karayigit et al., 2021; Robles-González et al., 2023; Parks et al., 2023; Snyder et al., 2024; Prather et al., 2024; Abumoh’d, 2024; Shlool and Abumoh’d, 2024; Ben Waer et al., 2021), permitting an outcome-specific reanalysis in which dependent effect sizes are modelled directly.

This review estimated the acute effects of caffeine ingestion on resistance exercise performance in women. We synthesized effects by outcome domain, maximal strength, muscular endurance, movement speed, functional strength, functional endurance, and total load rather than treating resistance exercise performance as a single construct. A three-level random-effects model with cluster-robust variance estimation was used to account for dependent effect sizes contributed by the same study. Dose, mean age, and resistance load were examined as exploratory study-level moderators where data were sufficient. The aim was to estimate outcome-specific effects and to assess whether current reporting of menstrual cycle phase, hormonal contraceptive use, habitual caffeine intake, and paired statistics is sufficient for female-specific inference.

## 2 Methods

### 2.1 Research protocol and registration

The protocol for this systematic review and meta-analysis was registered in the Open Science Framework on 28 April 2026 (OSF; https://osf.io/2msf6/overview; DOI: 10.17605/OSF.IO/2MSF6), and reporting follows the PRISMA 2020 statement (Page et al., 2021) in **Supplementary Document 1**. The database search had been completed on 26 April 2026 before registration. However, title/abstract screening, full-text eligibility assessment, data extraction, risk-of-bias assessment, certainty assessment, and statistical analysis had not begun at the time of registration. No deviations from the registered eligibility criteria, outcome domains, or planned synthesis structure were made after registration.

### 2.2 Inclusion and exclusion criteria

Literature screening followed a pre-specified PICOS framework. The population comprised women or mixed-sex cohorts from which female subgroup data could be independently extracted. The intervention was an acute caffeine ingestion administered as anhydrous caffeine, caffeine capsules, or caffeinated coffee; caffeine form was extracted and coded, and coffee-based trials were examined descriptively because blinding and non-caffeine constituents may differ from capsule trials. The comparator was a placebo or caffeine-free control matched for appearance, taste, capsule number or beverage volume, timing, and blinding procedures. Outcomes were resistance exercise performance indicators within six pre-defined domains: maximal strength, muscular endurance, movement speed, functional endurance, functional strength, and total load; functional outcomes were defined *a priori* as field-based performance tests involving resistance or strength-demanding actions rather than direct measures of external load, repetitions, velocity, or maximal force. Eligible designs were randomized placebo-controlled crossover or parallel-group trials; quasi-randomized studies were eligible only if allocation and sequence generation could be assessed. No language restrictions were applied at the search stage. We did not include conference abstracts, editorials, commentaries, or reviews because sufficient extractable paired outcome data and risk-of-bias information are usually unavailable in those formats; full-text peer-reviewed articles and online-first articles were eligible. Exclusion criteria were: (1) female subgroup data could not be separated from a mixed-sex sample; (2) caffeine was part of a multi-ingredient supplement and could not be isolated for an individual caffeine comparison; (3) the test task was not a resistance exercise performance outcome (e.g., only vascular outcomes were reported); and (4) the publication was a review, commentary, conference abstract, or editorial.

### 2.3 Search and screening strategy

**Figure 1:**
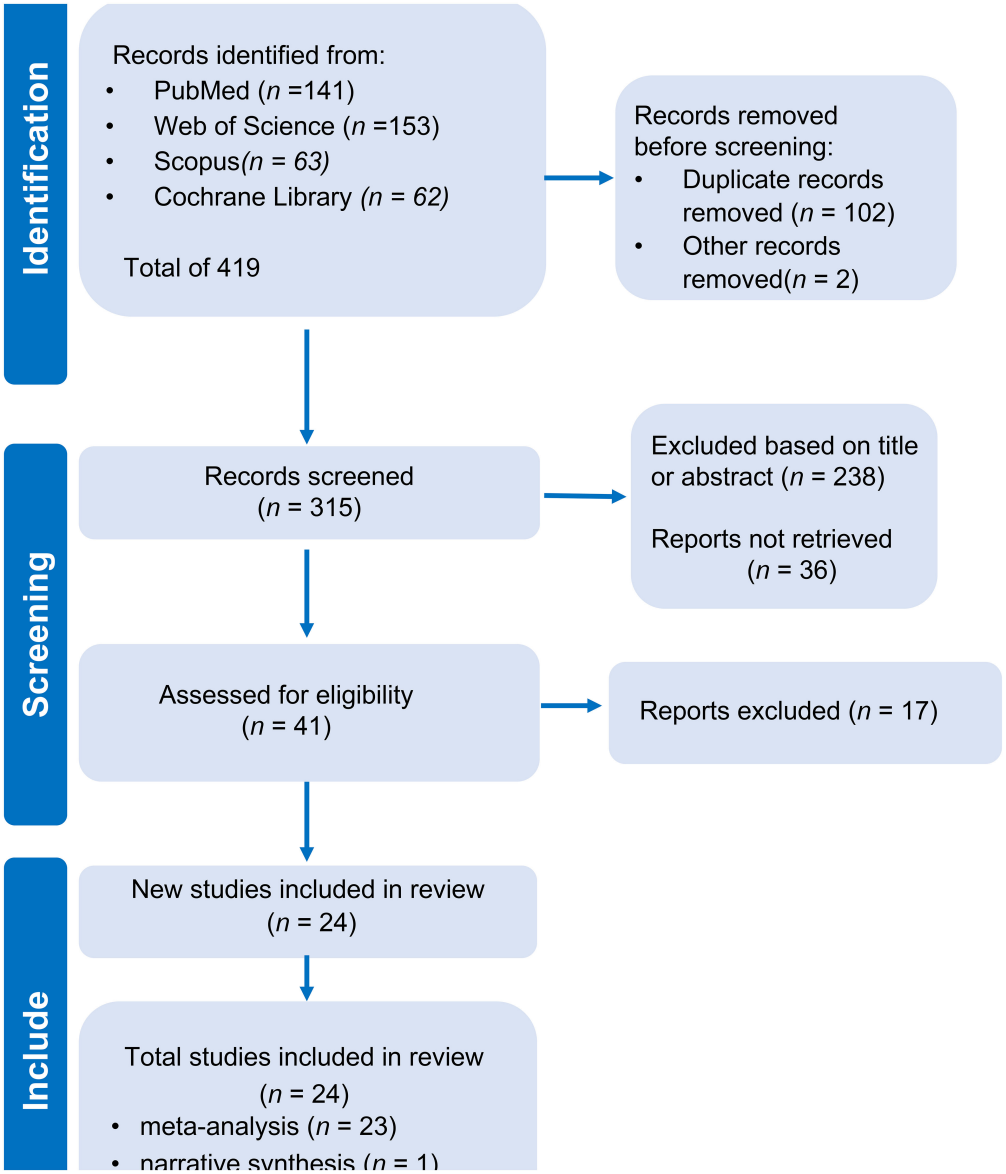
PRISMA 2020 study screening flowchart.

PubMed and Web of Science Core Collection were searched from database inception to 26 April 2026 using terms related to caffeine, female participants, resistance exercise, performance outcomes, and placebo-controlled trial designs. These two databases were selected because they index the biomedical, nutrition, and sports-science literature most directly relevant to placebo-controlled caffeine trials; the potential limitation of not searching additional databases is addressed in the Limitations section. The PubMed search strategy reported in the original manuscript has been corrected to match the registered protocol and retrieved 141 records; Web of Science Core Collection retrieved 153 records, the Scopus retrieved 63 records and the Cochrane Library retrieved 62 records. Complete database-specific search strings, final search dates, and records retrieved from each searched database are provided in **Supplementary Table 3**. Reference lists of relevant reviews and included studies were screened, and forward citation tracking was performed in Google Scholar/Web of Science; citation tracking identified no additional eligible studies beyond those captured by the database searches. Records were deduplicated manually in reference-management software and checked in Excel. No machine-learning or artificial-intelligence automation tools were used for deduplication, title/abstract screening, full-text screening, data extraction, or risk-of-bias assessment. After deduplication, two reviewers independently screened titles and abstracts against the PICOS criteria, assessed full texts for eligibility, extracted data, and evaluated risk of bias; disagreements were resolved by discussion or by consultation with a third reviewer. Scopus and Cochrane Library were additionally searched during revision using the same eligibility framework and an end-date limit of 26 April 2026 to maintain consistency with the registered search period. This additional search retrieved 125 records. After deduplication and screening, no additional eligible studies were identified beyond those already included (**Figure 1**).

### 2.4 Data extraction

For each included study, the following information was extracted: study design, participant characteristics (sample size, mean age, training status), caffeine dosage and administration method, timing of intake, exercise protocol and test movements, body region (upper/lower limb), resistance load (%1RM), menstrual cycle phase and hormonal status reporting, and the mean and standard deviation of each outcome at each condition. Each effect was assigned to one of six pre-specified outcome domains (maximal strength, muscular endurance, movement speed, functional endurance, functional strength, total load). Most effects were extracted from tables or raw data; graphical data from four trials were digitized with WebPlotDigitizer by two reviewers independently, with discrepancies resolved by rechecking image resolution, axis calibration, scale limits, and plotted values (Fett et al., 2018; Pereira et al., 2021; Prather et al., 2024; Romero-Moraleda et al., 2019). WebPlotDigitizer is useful for recovering numerical data from published graphs when authors do not report exact values, but it can introduce error from image resolution, symbol overlap, axis scaling, and manual point placement. Therefore, digitized values were treated as approximations, and studies relying on graphical extraction were considered in sensitivity and influence analyses. For one trial (Santana et al., 2022), the reported Hedges’ g and 95% confidence interval were converted to the sampling variance used in the meta-analysis because condition-level means and standard deviations were not reported; this study was also examined in a sensitivity analysis.

### 2.5 Risk of bias and methodological quality assessment

Risk of bias was assessed with the Cochrane Risk of Bias 2 (RoB 2) tool. Because nearly all included randomized trials used crossover designs, the RoB 2 tool for randomized crossover trials was applied to all eligible crossover studies, including assessment of period and carryover effects; the standard RoB 2 framework was reserved only for any eligible parallel-group comparison. Domains covered the randomization process, deviations from the intended intervention, missing outcome data, measurement of the outcome, selection of the reported result, and, for crossover designs, period effects and carryover effects (Sterne et al., 2019). An overall judgement of “low risk,” “some concerns,” or “high risk” was assigned. Methodological quality was additionally summarized using a modified PEDro scale (out of 10). Because several PEDro items fit acute crossover nutrition trials poorly, PEDro scores are reported only as supplementary descriptive information and were not used to assess the certainty of the evidence (de Morton, 2009; Maher et al., 2003).

### 2.6 Statistical analysis

Effect sizes were calculated as Hedges’ g for paired designs, with positive values indicating a preference for caffeine. Outcomes for which lower values indicated a favorable response, such as perceived exertion or discomfort, were reverse-coded so that positive values consistently favored caffeine. Magnitudes were interpreted cautiously using conventional standardized mean difference anchors (<0.20 trivial to small, approximately 0.20 small, approximately 0.50 moderate, and approximately 0.80 large), but practical interpretation also considered the 95% CI, prediction interval, test reliability, outcome domain, and GRADE certainty rather than relying on statistical significance alone. For crossover and repeated-measures designs, the standardized mean difference and its variance were computed following Morris and DeShon (2002), with a small-sample (Hedges’ g) correction. For crossover trials, paired standard deviations, change-score standard deviations, or within-participant correlations were extracted when available; when the within-participant correlation was unavailable, a value of 0.50 was imputed to compute the paired sampling variance because it represents a neutral mid-range assumption commonly used when paired correlations are unavailable, and sensitivity analyses used 0.25 and 0.75 to evaluate lower and higher plausible correlations. This imputed correlation was distinguished from the working covariance used to model dependence among multiple effect sizes from the same study. All analyses were performed in R using the metafor package (Viechtbauer, 2010), and cluster-robust variance estimation was used to handle effect-size dependence. Unless otherwise specified, tests were two-tailed.

Because most included studies used crossover designs, in which a single study can contribute dependent effect sizes across multiple doses, time points, or actions—violating the assumption of independent effect sizes—primary syntheses were conducted separately within each outcome domain using three-level random-effects models (the rma.mv function in metafor), with effect sizes nested within studies. These models partition sampling error (level 1), within-study heterogeneity among effect sizes from the same study (level 2), and between-study heterogeneity (level 3), using random intercepts for study and for effect size within study (Assink and Wibbelink, 2016; Cheung, 2014). Cluster-robust variance estimation with the CR2 correction and Satterthwaite degrees of freedom was used for all confidence intervals and tests of pooled effects and meta-regression coefficients (Pustejovsky and Tipton, 2022; Tipton, 2015). Heterogeneity was interpreted from the estimated variance components (τ² at level 2 and level 3), level-specific I² values where identifiable, total I², and prediction intervals. Level 2 I² was interpreted as the proportion of variability attributable to differences among effects within the same study cluster, whereas level 3 I² was interpreted as between-study heterogeneity. Cochran’s Q was treated descriptively because its interpretation is limited under dependent effect sizes (Higgins et al., 2003). Prediction intervals were calculated when the heterogeneity variance could be estimated, following IntHout et al. (2016), but were interpreted cautiously and were not used inferentially in sparse domains (Higgins et al., 2009). The six outcome domains were synthesized independently and were not collapsed into a single construct. Domains represented by only one or two independent study clusters were summarized descriptively rather than treated as stable pooled estimates: functional endurance (1 cluster) and functional strength (1 cluster) were reported descriptively without random-effects pooling, and total load (2 clusters) was summarized descriptively because the between-study variance was not identifiable.

Planned subgroup and continuous meta-regression analyses were conducted within outcome domains that had enough independent study clusters. Subgroup variables included caffeine dose (moderate dose 3 to <6 mg kg^−1^; high dose,≥6 mg·kg^1^ and body part (upper limb/lower limb). Menstrual cycle phase control was reported as a descriptive feature rather than a formal effect modifier in modeling because it is highly confounded with study, dose, and outcome constructs, and difficult to distinguish from physiological effects at the current reporting level (Sims and Heather, 2018; Elliott-Sale et al., 2021). Continuous moderating factors included caffeine dose, mean age of participants, and resistance load (%1RM). All moderator analyses were exploratory because dose, data source, outcome domain, training status, intake timing, and study design were not independently distributed across study clusters; inferences used the CR2 correction.

Contour-enhanced funnel plots were produced for outcome domains containing at least five effect sizes, and Egger regression intercepts were computed. Because effect sizes within studies are dependent and the number of independent study clusters was small, small-study effects were not formally tested when fewer than ten independent study clusters were available; funnel plots, where shown, were interpreted descriptively only (Sterne et al., 2011). Pre-specified sensitivity analyses included: (1) setting the within-study correlation to r = 0.25, 0.50, and 0.75 and refitting the models to test whether the direction of the pooled effect depended on the assumed paired correlation (Morris and DeShon, 2002); (2) leave-one-cluster-out analysis to identify influential clusters; (3) for the maximal strength domain, Harty et al. (2020) contributed three intake arms (30, 60, and 120 min before exercise) within the same cluster, so the primary model included only the 60-min arm and sensitivity analyses substituted the 30-min and 120-min arms; and (4) the maximal strength construct was limited to 1RM and isometric maximal voluntary contraction (MVC) in the primary model, with isokinetic peak torque analyzed as a separate sensitivity stratum. *Post-hoc* power calculations were not used to interpret null findings or to inform the certainty of evidence; imprecision was assessed through confidence intervals, prediction intervals, and GRADE (Greenland, 2012; Hoenig and Heisey, 2001).

The GRADE method was used to assess the certainty of evidence for each outcome domain, downgrading it based on risk of bias, inconsistency, indirectness, imprecision, and publication bias, ultimately categorizing the certainty of evidence as high, moderate, low, or very low (Guyatt et al., 2008). Domain-level judgments for each GRADE component are reported in **Supplementary Table 4**.

## 3 Results

### 3.1 Literature screening

**Figure 2:**
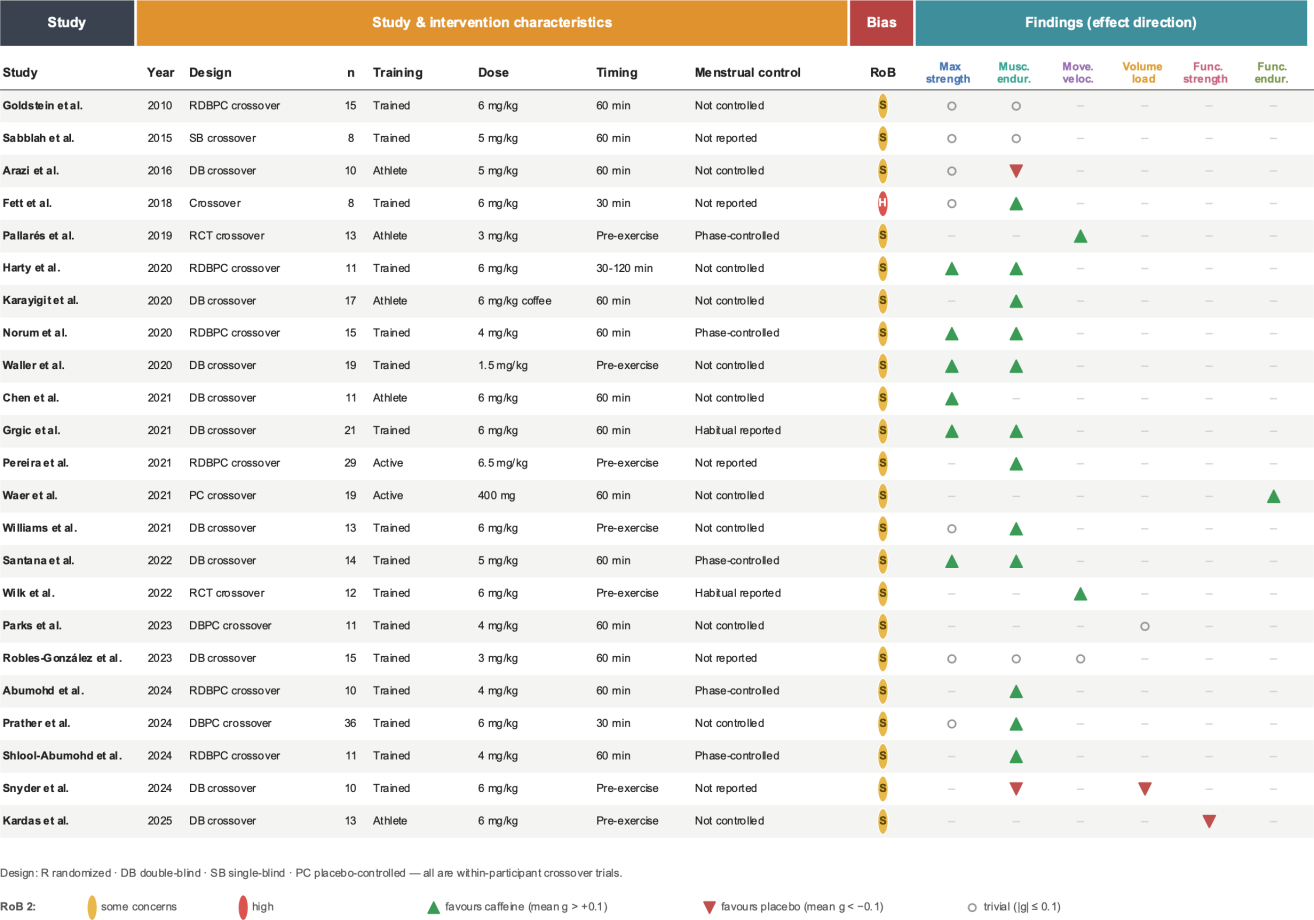
Summary of features and effect directions of included studies. Each study is listed with its design, training status, caffeine dosage, timing of intake, and menstrual cycle control. The right side uses directional markers to summarize the effect directions of each outcome domain (maximal strength, muscular endurance, movement speed, total load, functional strength, and functional endurance).

A combined database search retrieved 419 records (141 from PubMed, 153 from Web of Science Core Collection, 63 from Scopus and 62 from Cochrane Library). Citation tracking and reference-list checking were completed after database searching and did not add further eligible studies. Finally, 23 studies provided extractable effect sizes for the quantitative synthesis, while one study was retained only as a narrative background because key statistics could not be extracted (Kearney et al., 2025) (**Figure 2**).

### 3.2 Characteristics of included studies

Quantitative synthesis included 23 studies, and one additional study was retained only descriptively (Kearney et al., 2025). The included trials were predominantly acute, randomized, double-blind, placebo-controlled crossover studies in resistance- or strength-trained women (16 studies), athletes (5 studies), or healthy/physically active women (2 studies). Among the 18 studies reporting a numerical mean age, the mean age was 23.5 years (median 23.3; range of study-level means 19.7–31.0); the remaining samples spanned one adolescent cohort (Arazi et al., 2016) and one middle-aged cohort (Ben Waer et al., 2021). Most trials administered 3 to 6.5 mg·kg^-^¹ caffeine approximately 30 to 60 minutes before exercise; one trial delivered caffeine as caffeinated coffee at 3 and 6 mg·kg^-^¹ equivalents (Karayigit et al., 2020), one trial in middle-aged women used fixed doses of 100 mg and 400 mg (Ben Waer et al., 2021), and another varied intake timing (30, 60, and 120 minutes pre-exercise) at 6 mg·kg^-^¹ (Harty et al., 2020). Menstrual or hormonal status was controlled or standardized in 5 studies, whereas the remaining trials provided limited or unclear reporting. The number of effect sizes contributed per study cluster ranged from 1 to 24 (median 3): maximal strength comprised 13 clusters (33 effects), muscular endurance 17 clusters (35 effects), movement speed 3 clusters (30 effects), functional endurance 1 cluster (2 effects), functional strength 1 cluster (3 effects), and total load 2 clusters (4 effects).

### 3.3 Risk of bias and methodological quality

The RoB 2 domain-level assessments, the corresponding stacked summary chart, and the modified PEDro heatmap are presented in **Supplementary Document 3**. Of the 24 included study clusters (including the one descriptive cluster), none were judged at low overall risk of bias, 23 were judged to raise “some concerns,” and 1 was judged at high risk of bias. The high-risk judgement applied to Fett et al. (2018), mainly because its “baseline–caffeine–placebo–caffeine” dosing sequence made learning and carryover effects difficult to exclude (Sterne et al., 2019; Higgins et al., 2024b). The most common domain-level concerns were randomization reporting, handling of carryover effects, safeguards against selective reporting, and incomplete paired-data reporting. The modified PEDro score averaged 7.1 of 10 across the 24 clusters; 21 study clusters were rated good (≥7), 2 fair (4–6), and 1 poor (≤3). Because several PEDro items fit acute crossover nutrition trials poorly, this score is reported only as supplementary methodological information (de Morton, 2009; Maher et al., 2003).

### 3.4 Main analysis

**Figure 3:**
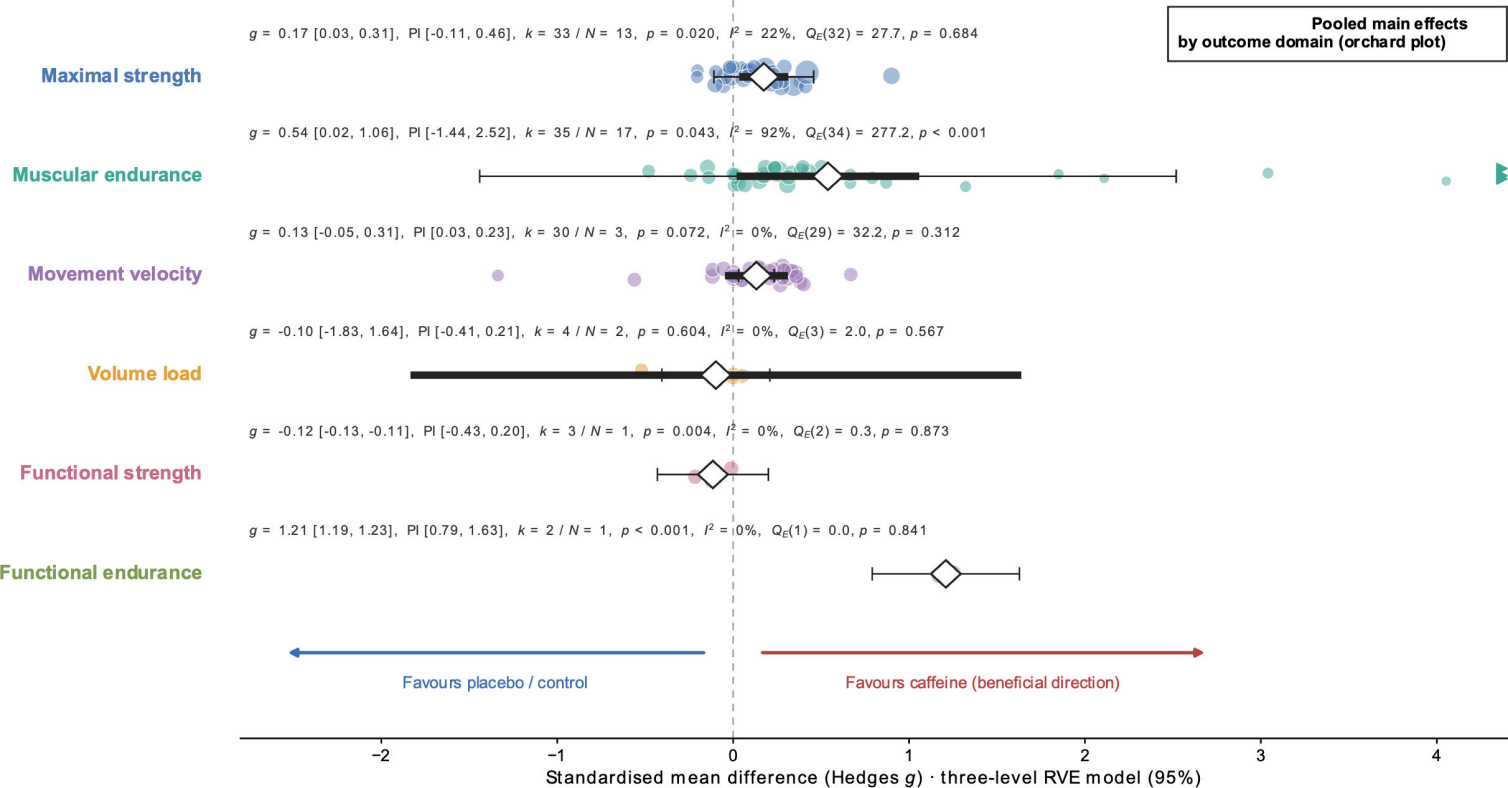
Orchard plot of pooled main effects for each outcome domain. Colored dots in each row represent individual effect estimates, with the area of the dot proportional to the precision (1/SE). Hollow white diamonds indicate pooled Hedges’ g for the three-level model; thick black bars represent the robust 95% confidence intervals for clustering, and thin black whiskers with caps represent the 95% prediction intervals. The vertical dashed line represents the zero-effect line, with a positive (right-hand) direction indicating a preference for caffeine. The figure labels k, number of studies N, p-value, total I², and, where identifiable, level-specific I² components for within-study (level 2) and between-study (level 3) heterogeneity.

The orchard plots summarizing the main effects of each outcome domain are shown in **Figure 3**.

**Figure 4:**
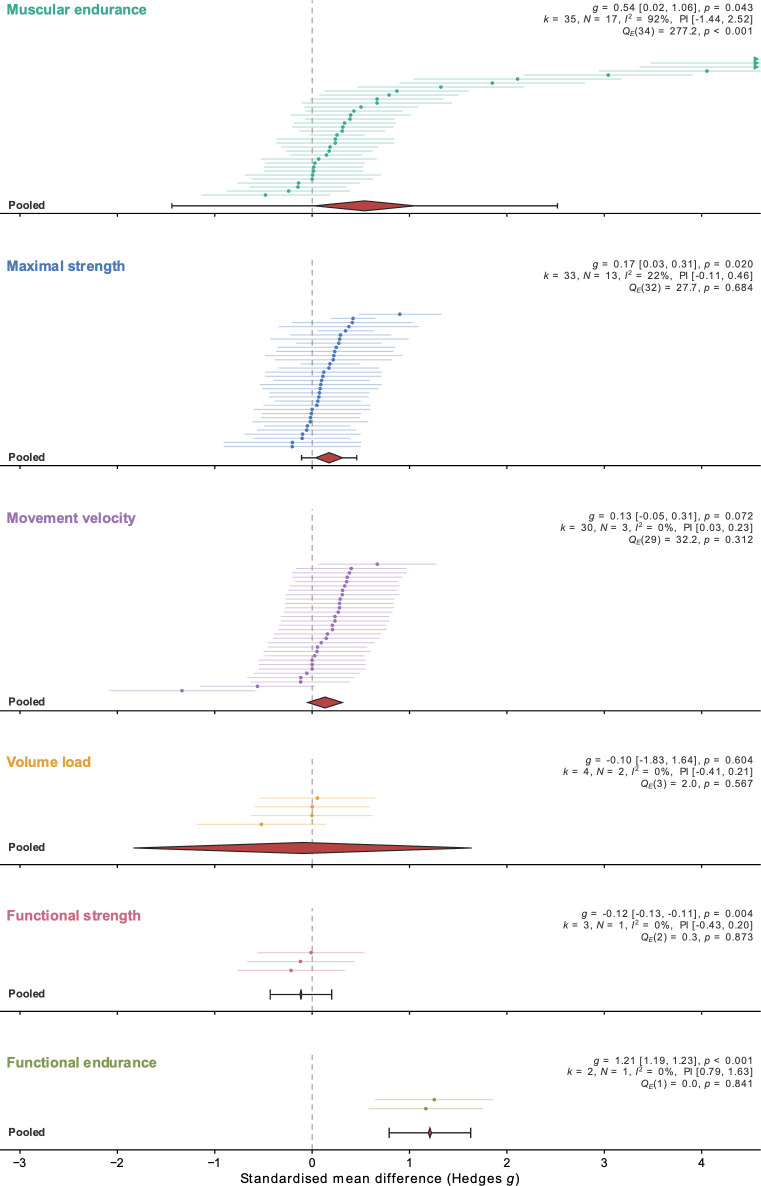
Caterpillar plot of all effect sizes arranged by outcome domain. Each thin line represents the 95% confidence interval for a single effect size, ordered by effect size; the red diamonds at the bottom of each domain represent the three-level pooled effect, and the black whiskers represent the 95% prediction interval; the vertical dashed line represents the zero-effect line, with a positive value indicating a favorable outcome for caffeine.

**Figure 5:**
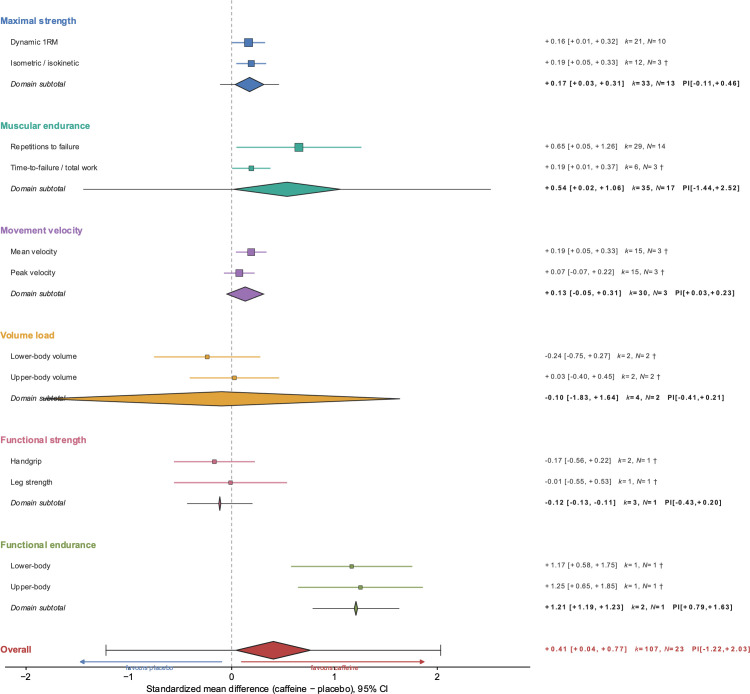
Synthetic forest plot of sub-outcomes within each outcome domain. Squares represent pooled estimates of each sub-outcome, their size proportional to precision; diamonds represent the subtotal of the outcome domain and the overall pooled effect (cluster robust CR2); thin lines represent the 95% prediction interval; k, number of studies, and 95% confidence interval are labeled for each estimate. Sub-outcomes containing only one study cluster or a single study are presented descriptively.

The caterpillar forest plot of all effect sizes for each outcome domain is shown in **Figure 4**, and the composite of sub-outcomes for each domain is shown in **Figure 5**. In the graphical representation, the two functional outcome domains and the total load are grouped under their parent categories (functional strength is grouped under strength, and total load and functional endurance are grouped under endurance), but all pooling, subgrouping, and meta-regression inferences maintain outcome domain specificity.

Compared with placebo, caffeine produced a small favorable effect on maximal strength (k = 33, studies = 13, g = 0.17, 95% CI 0.06 to 0.29; PI −0.11 to 0.46; CR2 p = 0.020; GRADE low). Heterogeneity was modest overall, but the prediction interval crossed zero, indicating that future comparable studies may show null effects. Muscular endurance showed a moderate average effect (k = 35, studies = 17, g = 0.54, 95% CI 0.06 to 1.02; PI −1.44 to 2.52; CR2 p = 0.043; GRADE low). Heterogeneity was substantial, with variability arising both within study clusters and between study protocols, and the wide prediction interval indicates low reproducibility across settings. Movement speed showed a small model-based estimate (k = 30, studies = 3, g = 0.13; model-based 95% CI 0.03 to 0.23), but level-specific heterogeneity could not be interpreted reliably because only three independent clusters were available and two clusters contributed many effects. The model-based and prediction intervals coincide because between-study heterogeneity was not separately identifiable in this sparse domain. Sub-outcome decompositions for each domain (e.g., 1RM and isometric/isokinetic strength within maximal strength, and repetitions to failure and time to failure/total work within muscular endurance) are shown in **Figure 5**.

### 3.5 Moderating factor analysis

**Figure 6:**
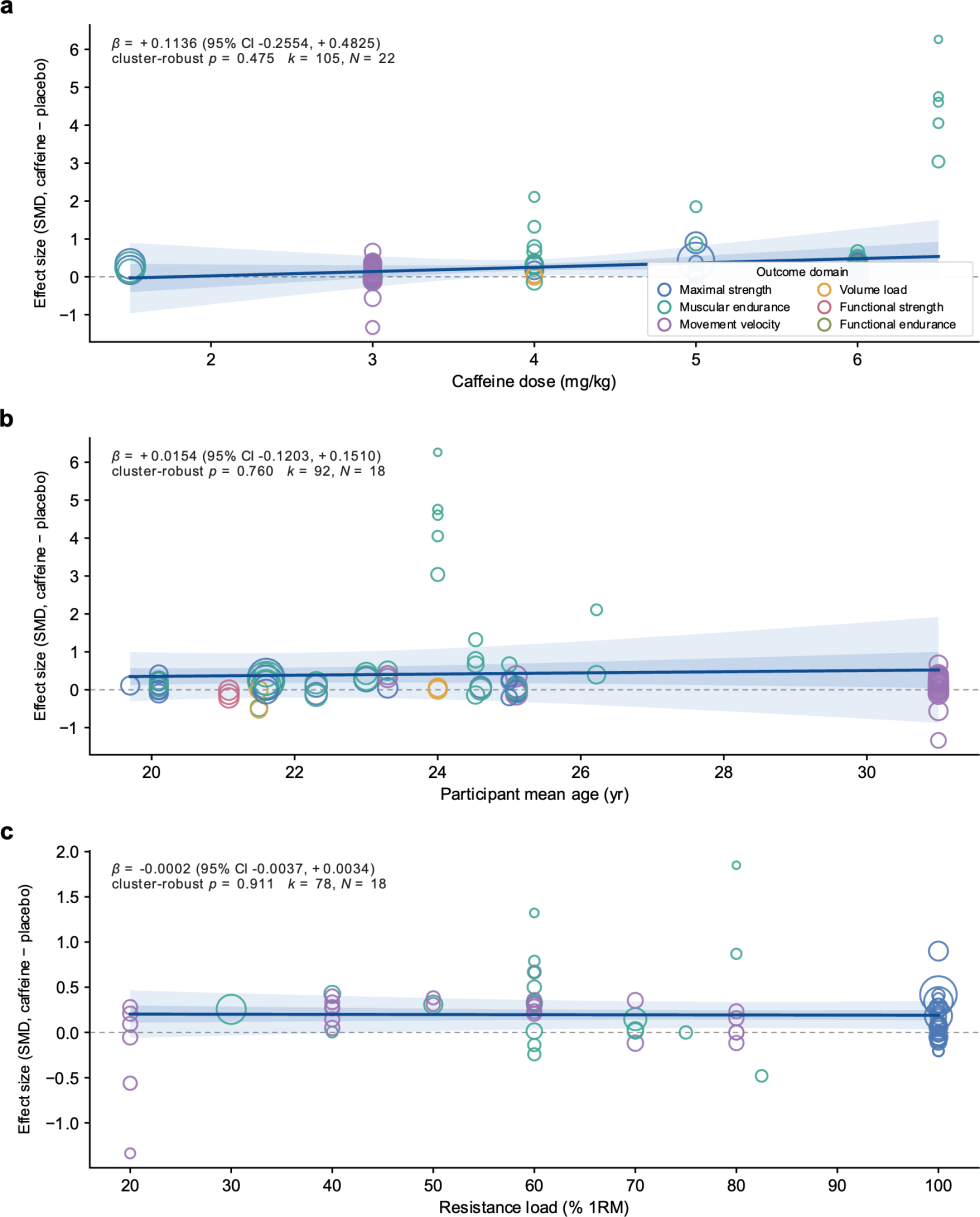
Exploratory three-level meta-regression bubble plot for continuous moderating factors. The three panels represent caffeine dose (mg·kg^-^¹), mean age of subjects (years), and resistance load (%1RM); bubble area is proportional to precision (1/SE), and bubble color represents the outcome domain; solid line represents cluster robust (CR2) regression fit, and shading represents its 95% confidence band. Moderating factors are confounded with outcome type, extraction source, and design; relationships are descriptive only and not causal.

The sub-outcome/sub-combination compositions and continuous meta-regression results for each outcome are visualized in **Figure 5** (sub-outcome forest plot) and **Figure 6** (meta-regression bubble plot), respectively. Given the partial confounding of dose, data source, outcome domain, training status, timing of caffeine intake, and study design among the 23 included clusters, all moderating factor results were treated as exploratory. Residual variance components and level-specific heterogeneity estimates were examined after moderator inclusion; moderators did not consistently reduce residual heterogeneity, so apparent subgroup differences should be interpreted as descriptive patterns rather than evidence that the moderators explain between-study variation.

#### 3.5.1 Maximal strength

Within the maximal strength domain, the g for the intermediate-dose subgroup (3 to <6 mg·kg^-^¹) was 0.22 (95% CI −0.02 to 0.46), and for the high-dose subgroup (≥6 mg·kg^-^¹) was 0.09 (95% CI −0.03 to 0.22). Lower-limb movements showed small, favorable estimates (g = 0.21, 95% CI 0.02 to 0.40), whereas upper-limb movements showed negligible estimates (g = 0.07, 95% CI −0.04 to 0.17). Menstrual cycle phase was treated as a descriptive study characteristic rather than a formal effect modifier (see Statistical Analysis). Point estimates differed numerically between trials with clearly reported phase-controlled tests (4 effects, 2 clusters) and trials with unclear or missing phase reporting (29 effects, 11 clusters); however, these strata were confounded with study, dose, and outcome construct, so the comparison reflects the heterogeneity of female-specific reporting more than any physiological effect of cycle phase. Within the maximal strength domain, continuous regression provided no convincing evidence of moderation by caffeine dose (β = −0.018, SE = 0.024, CR2 p = 0.516, k = 33, studies = 13) or mean age (β = −0.022, SE = 0.015, CR2 p = 0.235, k = 26, studies = 9); resistance load could not be modeled because all relevant effects were coded as 100% 1RM.

#### 3.5.2 Muscle endurance

Within the muscle endurance domain, the high-dose subgroup (≥6 mg·kg^-^¹) had a g = 0.68 (95% CI −0.18 to 1.54), and the intermediate-dose subgroup (3 to <6 mg·kg^-^¹) had a g = 0.47 (95% CI 0.10 to 0.85). The uncertainty in the high-dose estimate was greater. Lower limb and upper limb protocols were similar in direction (lower limb g = 0.68, 17 effects, 11 clusters; upper limb g = 0.56, 14 effects, 9 clusters), with overlapping confidence intervals. Trials controlling for menstrual cycle phase had a g = 0.76 (10 effects, 4 clusters), while trials with unclear or missing phase control had a g = 0.47 (25 effects, 13 clusters). Continuous regressions provided no reliable evidence of effect modification for caffeine dose (β = 0.173, SE = 0.190, CR2 p = 0.418, k = 35, number of studies = 17), mean age of participants (β = 0.101, SE = 0.093, CR2 p = 0.325, k = 29, number of studies = 13), or resistance load (β = −0.001, SE = 0.004, CR2 p = 0.771, k = 20, number of studies = 12).

#### 3.5.3 Movement speed

Within the movement speed domain, regressions of mean age and resistance load were based on only three study clusters, two of which contributed many within-study effect sizes (Romero-Moraleda et al., 2019; Filip-Stachnik et al., 2022; Robles-González et al., 2023). Neither moderator was statistically significant (age β = −0.008, SE = 0.020, CR² p = 0.764, k = 30, studies = 3; load β = 0.002, SE = 0.003, CR² p = 0.567, k = 28, studies = 3). These results are exploratory.

#### 3.5.4 Overall continuous moderating factors

Across all eligible effects, none of the three continuous moderators showed reliable evidence of effect modification: caffeine dose β = 0.114, SE = 0.149, CR2 p = 0.475 (k = 105, studies = 22); mean age β = 0.015, SE = 0.047, CR2 p = 0.760 (k = 92, studies = 18); resistance load β = 0.000, SE = 0.001, CR2 p = 0.911 (k = 78, studies = 18). Because the outcome domains were not interchangeable, these cross-domain regressions are reported only as descriptive diagnostics adjusted for outcome domain and should not be interpreted as pooled performance effects. The three-level meta-regression fits and bubble plots are shown in **Figure 6**.

### 3.6 Sensitivity analysis and precision

**Figure 7:**
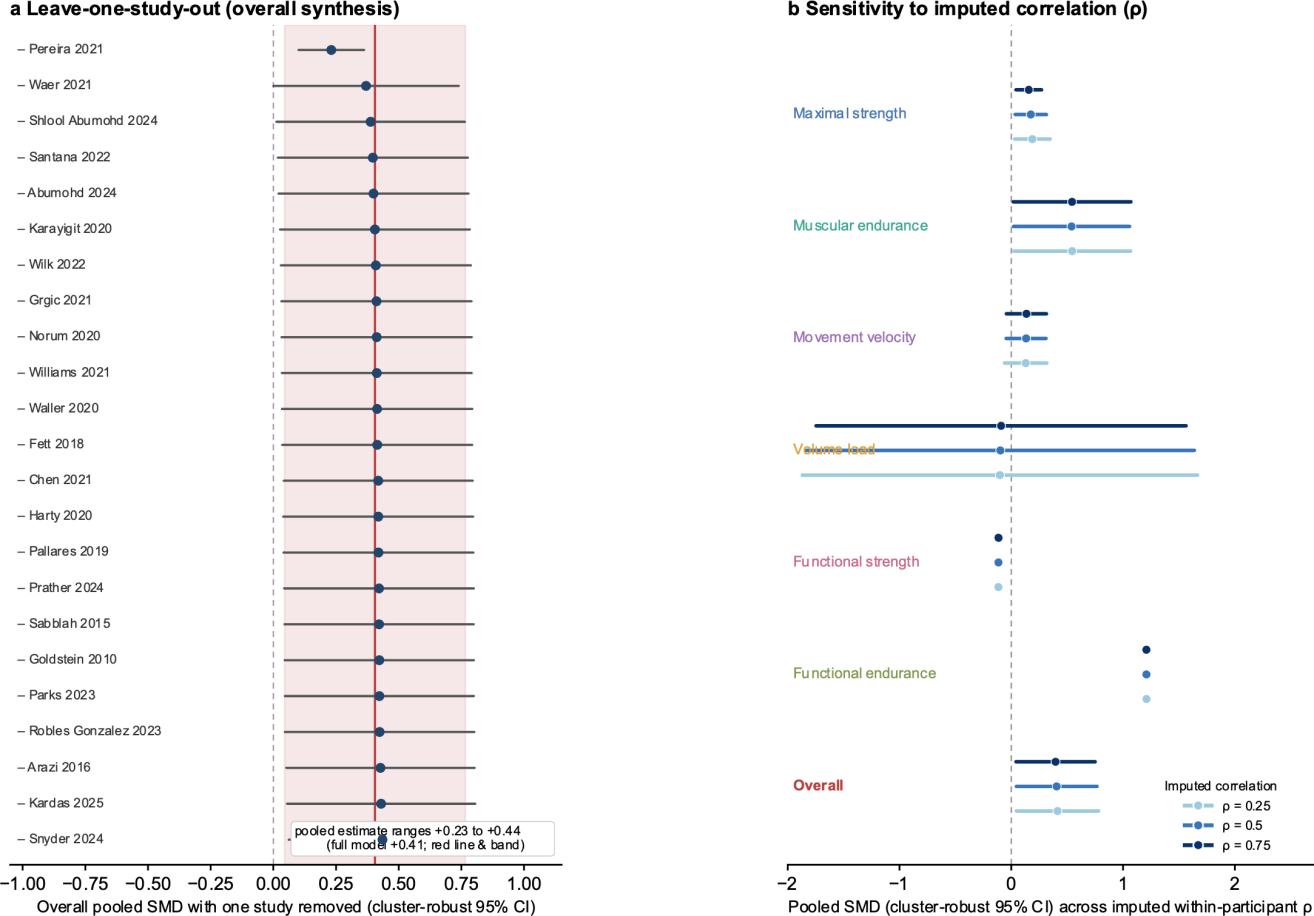
Sensitivity analysis. **(a)** Leave-one-cluster-out analyses for the main outcome domains, including the influence of the Pereira et al. (2021) cluster on muscular endurance; **(b)** sensitivity to the imputed within-participant correlation coefficient, showing that domain-specific estimates remained broadly stable across r = 0.25, 0.50, and 0.75.

When the imputed within-participant correlation was varied across r = 0.25, 0.50, and 0.75, domain-specific pooled estimates were stable in direction and magnitude, indicating that the pooled effects were not driven by the assumed paired correlation (Morris and DeShon, 2002). Sensitivity analyses were conducted separately within each outcome domain, and no overall cross-domain composite was used for inference. Leave-one-cluster-out analysis identified Pereira et al. (2021) as the most influential cluster for the muscular endurance estimate (**Figure 7a**).

**Figure 8:**
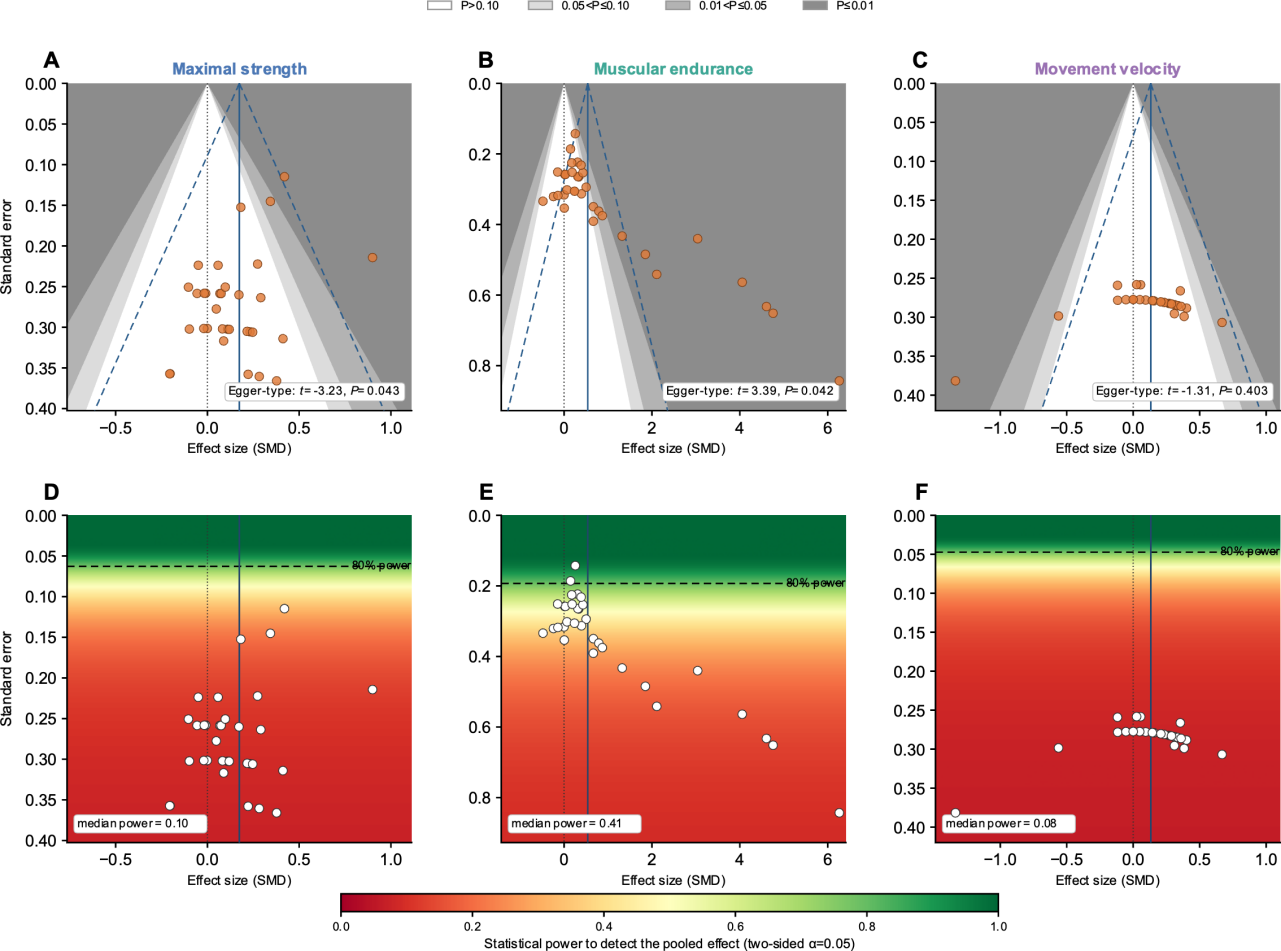
Funnel plots and precision diagnostics for the primary outcomes. The top row **(A-C)** shows contour-enhanced funnel plots for maximal strength, muscular endurance, and movement speed, with gray levels representing effect-significance contours and Egger regression intercepts labeled. The bottom row **(D-F)** shows the corresponding sunset-enhanced funnel plots, with colors indicating statistical power for detecting pooled effects and median power values (approximately 0.10, 0.41, and 0.08, respectively). Due to within-study dependence and sparse study clusters, the funnel plots are interpreted descriptively only.

Removing the Pereira et al. (2021) cluster attenuated the muscular endurance estimate, consistent with its inclusion of large effects from graphical digitization. Removing any other single cluster did not substantially change the direction or magnitude of the maximal strength or movement speed estimates. The three-level model including all effects was retained. **Figure 8** shows contour-enhanced funnel plots, Egger regression intercepts, and descriptive precision diagnostics for each main outcome (Hoenig and Heisey, 2001; Greenland, 2012).

### 3.7 Certainty of evidence

GRADE certainty of evidence (**Table 1**; **Supplementary Table 4**) was low for maximal strength, for risk of bias and imprecision; low for muscular endurance in the summary table but interpreted cautiously because inconsistency, wide prediction intervals, and influence diagnostics reduced confidence in reproducibility; and very low for movement speed, functional endurance, functional strength, and total load, owing to sparse study clusters, protocol indirectness, and imprecision (Guyatt et al., 2008). These judgments were aligned with the cautious interpretation used in the Discussion and Conclusion.

**Table 1:** Summary of GRADE certainty of evidence for each outcome domain.

Outcome	Number of studies/Number of effects	Effect(95% CI;PI)	GRADE	Interpretation
Maximal strength	13/33	0.17(0.06 to 0.29) PI −0.11 to 0.46	Low	Caffeine may have a slight beneficial effect on women’s maximum strength; however, the actual effect is limited.
Muscular endurance	17/35	0.54(0.06 to 1.02) PI −1.44 to 2.52	Low	Caffeine may improve muscle endurance, but the expected effects are highly variable and uncertain.
Movement speed	3/30	0.13(0.03 to 0.23) PI 0.03 to 0.23	Very low	There may be a slight, favorable velocity effect, but this is based on only three research clusters.
Functional endurance	1/2	1.21(0.79 to 1.63)	Very low	Observed in a single trial involving middle-aged women; not suitable for generalization.
Functional strength	1/3	−0.12(−0.43 to 0.20)	Very low	No clear benefit was observed in the individual female soccer player trial.
Total load	2/4	−0.10(−0.41 to 0.21)	Very low	No clear total load effect was observed.

## 4 Discussion

The central finding is not that caffeine uniformly improves resistance exercise performance in women, but that the certainty of evidence differs sharply by outcome domain. Maximal strength showed a small average effect (g = 0.17), whereas muscular endurance showed a larger but less reproducible average effect (g = 0.54) with substantial uncertainty. Evidence for movement speed, functional outcomes, and total load was restricted to a few independent study clusters. Continuous meta-regressions of caffeine dosage, mean age of participants, and resistance load did not provide reliable evidence that these factors explained effect variation, but these analyses were underpowered and confounded by outcome type, protocol, training status, and caffeine timing. These results should therefore be read as a cautious map of the current evidence base rather than as a basis for strong applied recommendations.

### 4.1 Maximal strength

The maximal strength effect in this review (g = 0.17, 95% CI 0.06 to 0.29; GRADE low) was smaller than pooled estimates of caffeine for resistance exercise in mixed-sex meta-analyses and similar to the most relevant previous women-specific review, which included fewer trials and used a traditional independent-effects random-effects model (Grgic et al., 2018; Grgic and Del Coso, 2021; Warren et al., 2010). Although statistically significant, the estimate is small and the prediction interval included zero; therefore, statistical significance should not be equated with practical importance for elite or highly trained athletes, where test reliability and the smallest worthwhile change are critical. Three factors may partly explain these differences. First, this review restricted inference to female subjects or independently extractable female subgroups, whereas mixed-sex pooling reduces the visibility of women-specific uncertainty (Goldstein et al., 2010, 2010; Guest et al., 2021; Sabblah et al., 2015). Second, multiple effects from the same crossover trial were modeled as nested within the study rather than as independent, which increased the standard error and widened the prediction interval (Hedges et al., 2010; Pustejovsky and Tipton, 2022). Third, each outcome was synthesized separately by domain rather than merged into a single strength-endurance construct. A small maximal strength effect is biologically plausible (Pickering and Grgic, 2019), but the present evidence is better interpreted as indicating a possible modest acute benefit than a reliable practical advantage across women or testing contexts.

### 4.2 Muscular endurance

The muscular endurance estimate was larger than that for maximal strength (g = 0.54 vs. g = 0.17). Although the CR2-adjusted confidence interval excluded zero (95% CI 0.06 to 1.02; CR2 p = 0.043), three points warrant caution. First, excluding Pereira et al. (2021), a cluster digitized from graphs and containing several large positive effects from a 6.5 mg·kg^-^¹ protocol, weakened the pooled estimate (Pereira et al., 2021). Second, the prediction interval spanned negative and positive values, indicating that future comparable protocols could show smaller, null, or unfavorable effects (IntHout et al., 2016). Third, the high-dose estimate was more uncertain than the moderate-dose estimate, which is more consistent with protocol variability than with a confirmed dose-response pattern. This pattern aligns with the broader caffeine-resistance literature, in which effects on muscular endurance are often larger than on maximal strength but also more heterogeneous (Ferreira et al., 2021; Grgic et al., 2018; Polito et al., 2016; Warren et al., 2010). A mechanism centered on central drivers of perceived effort, pain, and fatigue predicts a larger effect on repeated submaximal contractions than on single maximal efforts (Davis et al., 2012; Doherty and Smith, 2005). Early-follicular-phase trials provide relevant endurance data (Abumoh’d, 2024; Shlool and Abumoh’d, 2024), but female-specific evidence remains insufficient to support strong generalization about muscular endurance benefits. The tension between the positive mean estimate and the wide prediction interval is central to interpretation: caffeine may benefit some muscular endurance protocols, but the existing literature has not identified the conditions under which this benefit is reproducible, so the pooled g = 0.54 should be read as an average rather than a transferable effect.

### 4.3 Movement speed, functional outcomes, and total load

The movement speed estimate is consistent with previous reports that caffeine has a small acute effect on barbell velocity in trained individuals (Raya-González et al., 2020; Robles-González et al., 2023; Wilk et al., 2019, 2019). However, the female-specific estimate (g = 0.13) was based on only three study clusters (Filip-Stachnik et al., 2022; Robles-González et al., 2023; Romero-Moraleda et al., 2019), two of which contributed many within-study effects. For movement speed, this apparent precision is driven by many effects extracted within a few protocols rather than by replication across independent samples, which explains the coexistence of a narrow confidence interval and very low certainty; the CR2 inference returned p = 0.072. This finding should therefore be interpreted as preliminary and cluster-limited, not as evidence sufficient to support strong conclusions or practical recommendations (Pustejovsky and Tipton, 2022). Functional endurance was based on a single trial in middle-aged Tunisian women at fixed doses of 100 mg and 400 mg (Ben Waer et al., 2021), and functional strength on a single trial in female soccer players (Mor et al., 2025). These domains are retained for completeness but should be read as single-cluster signals; they are more accurately described as insufficient evidence than as preliminary positive or negative results, and neither can support application-level recommendations. Total load did not show a clear effect (g = −0.10, 95% CI −1.83 to 1.64) (Parks et al., 2023; Snyder et al., 2024).

### 4.4 Moderating factors: caffeine dosage, age, and resistance load

Study-level moderator analyses did not provide reliable evidence that caffeine dose, mean age, or resistance load explained between-study variation, and residual heterogeneity was not consistently reduced after including these moderators. These variables should be interpreted as moderators that were only weakly explorable with the current evidence base, rather than as confirmed null moderators. The dose range was narrow, mostly 3 to 6.5 mg·kg^-^¹ and clustered around 3 to 6 mg·kg^-^¹ (Guest et al., 2021; Maughan et al., 2018). Mean age was also restricted, with most participants aged 18 to 30 years and only one trial in middle-aged women (Ben Waer et al., 2021). Study level mean age is a poor proxy for reproductive hormone status because women of the same chronological age may differ in menstrual cycle phase, hormonal contraceptive use, endocrine profile, perimenopausal status, and menopausal transition. Resistance load was also difficult to interpret as an independent moderator because it was partly confounded with the outcome domain: maximal strength was coded as 100% 1RM by definition, whereas muscular endurance tests typically used approximately 60% to 70% of 1RM. Caffeine timing was systematically varied in only one trial (Harty et al., 2020), further limiting comparative inference. A second tier of moderators is theoretically central but insufficiently reported or inconsistently controlled in the available studies. These include menstrual cycle phase, hormonal contraceptive use, menopausal status, habitual caffeine intake, and recent caffeine withdrawal. The inconsistent reporting of menstrual cycle phase and hormonal contraceptive use should be regarded not only as a limitation of this review but also as a major field-level gap that currently prevents female-specific individualized recommendations.

### 4.5 Publication bias and small-study effects

Egger regression and contour-enhanced funnel plots suggested possible small-study effects for maximal strength and muscular endurance. These diagnostics should be interpreted cautiously because effect sizes were dependent, the number of independent study clusters was limited, and several studies contributed multiple effects from the same participants. Nevertheless, possible small-study effects could mean that the pooled estimates for maximal strength and muscular endurance are somewhat inflated if smaller studies with favorable findings are more likely to be published or reported. This concern is especially relevant for muscular endurance, where the pooled estimate was influenced by a limited number of large positive effects and the prediction interval was very wide. We therefore interpret publication-bias analyses as exploratory and emphasize the direction, magnitude, prediction intervals, influence diagnostics, and GRADE certainty together rather than treating the pooled effects as definitive estimates.

### 4.6 Practical implications and future research

Current evidence does not justify a general claim that acute caffeine ingestion improves resistance exercise performance in women. The available data suggest that any ergogenic effect is outcome specific, context dependent, and likely smaller than implied by mixed sex evidence. In trained women who already tolerate caffeine, maximal strength may improve acutely, but the average effect appears modest and should be interpreted against test reliability, biological variability, and the smallest performance change that would matter in practice. Muscular endurance may be more responsive in some protocols, particularly when sets are performed close to failure, but this finding should not be generalized across exercises, loading schemes, training backgrounds, or caffeine doses without further replication (Grgic and Del Coso, 2021). In contrast, evidence remains insufficient for applying caffeine to improve movement velocity, functional resistance exercise outcomes, or total training volume in women. Therefore, caffeine should be considered an individualized acute strategy rather than a routine recommendation. Practical use should account for habitual caffeine intake, prior response, gastrointestinal symptoms, anxiety, sleep disruption, training time, and the consequences of impaired recovery after evening ingestion (Burke, 2008; Guest et al., 2021; Maughan et al., 2018; Spriet, 2014).

**Figure 9:**
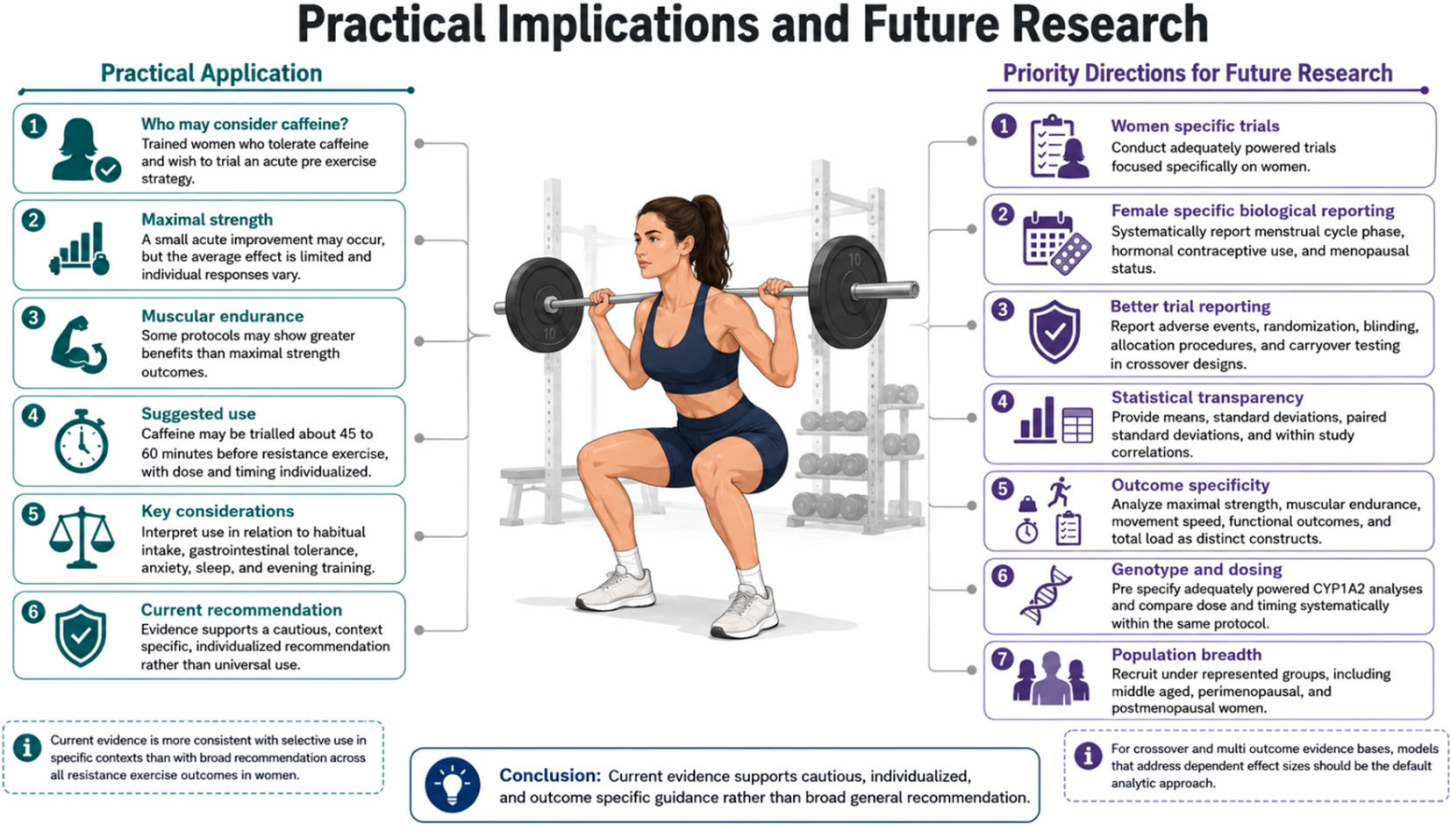
Practical recommendations and future priorities based on current evidence.

Future research should move beyond asking whether caffeine is ergogenic in women and instead define for whom, under what conditions, and for which resistance exercise outcomes caffeine is likely to be useful. Adequately powered trials designed specifically for women are needed, with explicit reporting of menstrual cycle phase, hormonal contraceptive use, menopausal status, habitual caffeine intake, sleep, adverse symptoms, and prior caffeine responsiveness (Costello et al., 2014; Romero-Moraleda et al., 2019; Elliott-Sale et al., 2021). Trial reporting should include randomization, allocation sequence, blinding integrity, condition order, washout duration, and tests for period or carryover effects, because much of the current evidence depends on crossover designs (Elbourne et al., 2002; Sterne et al., 2019). Statistical reporting also needs improvement. Studies should provide condition level means and standard deviations, change scores where appropriate, paired standard deviations, within participant correlations, and preferably individual participant data, because these values are required to model dependent effects and within person heterogeneity correctly (Morris and DeShon, 2002; Hedges et al., 2010). Future reviews should avoid treating maximal strength, muscular endurance, movement velocity, functional performance, and total load as interchangeable indices of a single resistance performance construct unless that decision is explicitly justified as exploratory (Grgic et al., 2018; Higgins et al., 2024a). Genotype analyses, especially CYP1A2, should be prespecified and powered as effect modification analyses rather than added *post hoc* (Prather et al., 2024). Dose and timing should be compared within the same protocol and population, because indirect comparisons across heterogeneous studies cannot separate pharmacological effects from differences in exercise task, training status, and measurement error (Southward et al., 2018). Finally, recruitment should extend beyond young trained women to include middle aged, perimenopausal, and postmenopausal women, since endocrine status, sleep vulnerability, muscle function, and cardiometabolic risk may alter both the benefit and the cost of caffeine use (Costello et al., 2014; Cowley et al., 2021; Elliott-Sale et al., 2021) (**Figure 9**).

### 4.7 Strengths and limitations

This review addressed several methodological issues that are common in the caffeine and resistance exercise literature. The synthesis was restricted to female participants or independently extractable female subgroups, which avoided mixed sex pooling and preserved women-specific uncertainty (Guest et al., 2021; Warren et al., 2010; Grgic and Del Coso, 2021). The six resistance exercise outcome domains were analyzed separately, reducing the risk of combining physiologically distinct constructs such as maximal strength, muscular endurance, power, and movement velocity (Grgic et al., 2018; Higgins et al., 2024a). In addition, the use of a three-level random effects model with cluster robust CR2 variance estimation, Satterthwaite degrees of freedom, and prediction intervals accounted for dependence among multiple effects from the same study and reduced the risk of overly precise estimates (Hedges et al., 2010; IntHout et al., 2016; Pustejovsky and Tipton, 2022; Tipton, 2015). Sensitivity analyses using imputed within-study correlations of 0.25, 0.50, and 0.75, together with leave-one-cluster-out analyses, showed that the direction of the main findings was not materially changed by these analytical choices (Morris and DeShon, 2002; Elbourne et al., 2002) (**Figure 7b**).

The main limitations were sparse independent study clusters, incomplete female-specific reporting, and the frequent absence of paired statistics. Only PubMed, Web of Science Core Collection, Scopus and Cochrane Library were searched, which may have missed eligible studies indexed only in databases such as SPORTDiscus, Embase, or PsycINFO; this limitation should be considered when interpreting the completeness of the evidence base, although citation tracking and reference-list checking did not identify additional eligible studies. Moderator analyses were exploratory because the number of independent clusters was limited, and several moderators were partly confounded with outcome domain, caffeine dose, training status, and study design. These results should therefore be interpreted as hypothesis-generating rather than causal evidence of effect modification. Although most included studies used crossover designs, many did not report paired standard deviations, within participant correlations, sequence allocation, washout adequacy, or tests for period and carryover effects. These reporting gaps required analytical assumptions and limited the precision of some estimates. Reporting of menstrual cycle phase, hormonal contraceptive use, menopausal status, and habitual caffeine intake was also inconsistent, which limits the extent to which pooled estimates can be applied to specific female subgroups, including adolescent athletes, perimenopausal and postmenopausal women, and habitual high caffeine consumers (Elliott-Sale et al., 2021; Pickering and Grgic, 2019; Romero-Moraleda et al., 2019).

## 5 Conclusion

Acute caffeine ingestion may produce a small improvement in maximal strength and may benefit muscular endurance in some women and protocols, but the certainty of evidence is low or very low for most outcomes and prediction intervals indicate limited reproducibility. Evidence for movement speed, functional outcomes, and total load remains sparse and should be considered preliminary or insufficient for application-level recommendations. Current data do not support strong outcome-specific recommendations based on dose, age, resistance load, menstrual cycle phase, hormonal status, or habitual caffeine intake. Future trials should prespecify primary resistance exercise outcomes and report paired statistics, menstrual cycle phase, hormonal contraceptive use, menopausal status, habitual caffeine intake, adverse symptoms, blinding integrity, and washout procedures.

## Data Availability

The original contributions presented in the study are included in the article/**Supplementary Material**. Further inquiries can be directed to the corresponding author.
