# Supplementary material for: Acute caffeine supplementation and resistance exercise performance in female participants: a systematic review and three-level meta-analysis
Source: Front Physiol. 2026 Jul 29;17:1892370. doi: 10.3389/fphys.2026.1892370 (PMC13461412; doi:10.3389/fphys.2026.1892370)
Supplement: Supplementary file 1 [file DataSheet1.docx]

SUPPLEMENTARY MATERIALS

| 、**Section and Topic** | **Item #** | **Checklist item** | **Location where item is reported** |
| --- | --- | --- | --- |
| **TITLE Acute Caffeine Supplementation and Resistance Exercise Performance in Female Participants: A Systematic Review and Three-Level Meta-Analysis** | | | Title |
| Title | 1 | Identify the report as a systematic review. | Title |
| **ABSTRACT** | | |  |
| Abstract | 2 | See the PRISMA 2020 for Abstracts checklist. | Abstract |
| **INTRODUCTION** | | |  |
| Rationale | 3 | Describe the rationale for the review in the context of existing knowledge. | Introduction |
| Objectives | 4 | Provide an explicit statement of the objective(s) or question(s) the review addresses. | Introduction |
| **METHODS** | | |  |
| Eligibility criteria | 5 | Specify the inclusion and exclusion criteria for the review and how studies were grouped for the syntheses. | Methods |
| Information sources | 6 | Specify all databases, registers, websites, organisations, reference lists and other sources searched or consulted to identify studies. Specify the date when each source was last searched or consulted. | Methods |
| Search strategy | 7 | Present the full search strategies for all databases, registers and websites, including any filters and limits used. | Methods |
| Selection process | 8 | Specify the methods used to decide whether a study met the inclusion criteria of the review, including how many reviewers screened each record and each report retrieved, whether they worked independently, and if applicable, details of automation tools used in the process. | Methods |
| Data collection process | 9 | Specify the methods used to collect data from reports, including how many reviewers collected data from each report, whether they worked independently, any processes for obtaining or confirming data from study investigators, and if applicable, details of automation tools used in the process. | Methods |
| Data items | 10a | List and define all outcomes for which data were sought. Specify whether all results that were compatible with each outcome domain in each study were sought (e.g. for all measures, time points, analyses), and if not, the methods used to decide which results to collect. | Methods |
|  | 10b | List and define all other variables for which data were sought (e.g. participant and intervention characteristics, funding sources). Describe any assumptions made about any missing or unclear information. | Methods |
| Study risk of bias assessment | 11 | Specify the methods used to assess risk of bias in the included studies, including details of the tool(s) used, how many reviewers assessed each study and whether they worked independently, and if applicable, details of automation tools used in the process. | Methods |
| Effect measures | 12 | Specify for each outcome the effect measure(s) (e.g. risk ratio, mean difference) used in the synthesis or presentation of results. | Methods |
| Synthesis methods | 13a | Describe the processes used to decide which studies were eligible for each synthesis (e.g. tabulating the study intervention characteristics and comparing against the planned groups for each synthesis (item #5)). | Methods |
|  | 13b | Describe any methods required to prepare the data for presentation or synthesis, such as handling of missing summary statistics, or data conversions. | Methods |
|  | 13c | Describe any methods used to tabulate or visually display results of individual studies and syntheses. | Methods |
|  | 13d | Describe any methods used to synthesize results and provide a rationale for the choice(s). If meta-analysis was performed, describe the model(s), method(s) to identify the presence and extent of statistical heterogeneity, and software package(s) used. | Methods |
|  | 13e | Describe any methods used to explore possible causes of heterogeneity among study results (e.g. subgroup analysis, meta-regression). | Methods |
|  | 13f | Describe any sensitivity analyses conducted to assess robustness of the synthesized results. | Methods |
| Reporting bias assessment | 14 | Describe any methods used to assess risk of bias due to missing results in a synthesis (arising from reporting biases). | Methods |
| Certainty assessment | 15 | Describe any methods used to assess certainty (or confidence) in the body of evidence for an outcome. | Methods |

**Supplementary Table S1. (continued).**

| **RESULTS** | | |  |
| --- | --- | --- | --- |
| Study selection | 16a | Describe the results of the search and selection process, from the number of records identified in the search to the number of studies included in the review, ideally using a flow diagram. | Results |
|  | 16b | Cite studies that might appear to meet the inclusion criteria, but which were excluded, and explain why they were excluded. | Results |
| Study characteristics | 17 | Cite each included study and present its characteristics. | Results |
| Risk of bias in studies | 18 | Present assessments of risk of bias for each included study. | Results |
| Results of individual studies | 19 | For all outcomes, present, for each study: (a) summary statistics for each group (where appropriate) and (b) an effect estimate and its precision (e.g. confidence/credible interval), ideally using structured tables or plots. | Results |
| Results of syntheses | 20a | For each synthesis, briefly summarise the characteristics and risk of bias among contributing studies. | Results |
|  | 20b | Present results of all statistical syntheses conducted. If meta-analysis was done, present for each the summary estimate and its precision (e.g. confidence/credible interval) and measures of statistical heterogeneity. If comparing groups, describe the direction of the effect. | Results |
|  | 20c | Present results of all investigations of possible causes of heterogeneity among study results. | Results |
|  | 20d | Present results of all sensitivity analyses conducted to assess the robustness of the synthesized results. | Results |
| Reporting biases | 21 | Present assessments of risk of bias due to missing results (arising from reporting biases) for each synthesis assessed. | Results |
| Certainty of evidence | 22 | Present assessments of certainty (or confidence) in the body of evidence for each outcome assessed. | Results |
| **DISCUSSION** | | |  |
| Discussion | 23a | Provide a general interpretation of the results in the context of other evidence. | Discussion |
|  | 23b | Discuss any limitations of the evidence included in the review. | Discussion |
|  | 23c | Discuss any limitations of the review processes used. | Discussion |
|  | 23d | Discuss implications of the results for practice, policy, and future research. | Discussion |
| **OTHER INFORMATION** | | |  |
| Registration and protocol | 24a | Provide registration information for the review, including register name and registration number, or state that the review was not registered. | Methods |
|  | 24b | Indicate where the review protocol can be accessed, or state that a protocol was not prepared. | Methods |
|  | 24c | Describe and explain any amendments to information provided at registration or in the protocol. | Methods |
| Support | 25 | Describe sources of financial or non-financial support for the review, and the role of the funders or sponsors in the review. | Funding |
| Competing interests | 26 | Declare any competing interests of review authors. | Conflict of Interest |
| Availability of data, code and other materials | 27 | Report which of the following are publicly available and where they can be found: template data collection forms; data extracted from included studies; data used for all analyses; analytic code; any other materials used in the review. | Data Availability Statement |

**Supplementary document 2. Characteristics of studies included in the quantitative synthesis**

| **Study (year)** | **Country** | **Sample** | **Training status** | **Age (y)** | **Design** | **Caffeine dose** | **Timing** | **Comparator** | **Menstrual control** | **Outcomes contributed (no. of effects)** |
| --- | --- | --- | --- | --- | --- | --- | --- | --- | --- | --- |
| **Goldstein (2010)** | United States | 15 women | resistance-trained | 24.6 | randomized double-blind placebo-controlled crossover | 6 mg/kg | 60 min pre-exercise | placebo | Not/unclear | 2 effects: Maximal strength (1); Muscular endurance (1) |
| **Sabblah (2015)** | United Kingdom | 8 women subgroup from 16 resistance-trained adults | moderately active resistance-trained | NR | single-blind placebo-controlled crossover | 5 mg/kg | 60 min pre-exercise | placebo | Not/unclear | 3 effects: Maximal strength (2); Muscular endurance (1) |
| **Arazi (2016)** | Iran | 10 teenage female karate athletes | karate athletes with at least 3 years experience | NR | double-blind randomized counterbalanced crossover | 2 mg/kg; 5 mg/kg | 60 min pre-exercise | placebo | Not/unclear | 2 effects: Maximal strength (1); Muscular endurance (1) |
| **Fett (2018)** | Brazil | 8 women | minimum 12 months continuous resistance training | 25 | crossover with basal caffeine placebo caffeine sequence | 6 mg/kg | 30 min pre-exercise | starch placebo | Not/unclear | 4 effects: Maximal strength (3); Muscular endurance (1) |
| **Pallares (2019)** | Spain | 13 eumenorrheic women | trained | 31 | randomized crossover | 3 mg/kg | pre-exercise | placebo | Controlled | 24 effects: Movement speed (24) |
| **Harty (2020)** | United States | 11 female subgroup from 29 healthy resistance-trained adults | resistance-trained | 20.1 | randomized double-blind placebo-controlled crossover | 6 mg/kg | 120, 60, or 30 min pre-exercise | placebo | Not/unclear | 12 effects: Maximal strength (9); Muscular endurance (3) |
| **Karayigit (2020)** | Turkey or multicentre setting | 17 caffeine-naive female athletes | team-sport or trained female athletes | 23 | randomized double-blind crossover | 3 and 6 mg/kg caffeinated coffee | 60 min pre-exercise | decaffeinated coffee | Not/unclear | 1 effects: Muscular endurance (1) |
| **Norum (2020)** | Norway | 15 women | resistance-trained | NR | randomized double-blind placebo-controlled crossover | 4 mg/kg | 60 min pre-exercise | placebo | Controlled | 4 effects: Maximal strength (2); Muscular endurance (2) |
| **Waller (2020)** | United States | 19 analyzable women | trained or recreationally trained | 21.6 | double-blind randomized crossover | 100 mg approximately 1.5 mg/kg | pre-exercise | placebo | Not/unclear | 4 effects: Maximal strength (2); Muscular endurance (2) |
| **Chen (2021)** | United States | 11 female collegiate athletes | trained athletes | 19.7 | randomized double-blind crossover | 6 mg/kg | 60 min pre-exercise | placebo | Not/unclear | 1 effects: Maximal strength (1) |
| **Grgic (2021)** | Australia or Serbia research setting | 21 women habituated to caffeine | resistance-trained | 23 | randomized double-blind crossover | 2 mg/kg; 4 mg/kg; 6 mg/kg | 60 min pre-exercise | placebo | Not/unclear | 2 effects: Maximal strength (1); Muscular endurance (1) |
| **Pereira (2021)** | Brazil | 29 women | physically active | 24 | randomized double-blind placebo-controlled crossover | 6.5 mg/kg | pre-exercise | placebo; carbohydrate mouth rinse; caffeine plus carbohydrate | Not/unclear | 5 effects: Muscular endurance (5) |
| **Ben Waer (2021)** | Tunisia | 19 healthy middle-aged women | healthy middle-aged | NR | placebo-controlled crossover | 100 mg; 400 mg | 60 min pre-exercise | placebo | Not/unclear | 2 effects: Functional endurance (2) |
| **Williams (2021)** | United States | 13 to 14 women | strength-trained | 23.3 | randomized double-blind crossover | 2 mg/kg; 4 mg/kg; 6 mg/kg | pre-exercise | placebo | Not/unclear | 2 effects: Maximal strength (1); Muscular endurance (1) |
| **Santana (2022)** | Brazil | 14 naturally menstruating resistance-trained women | resistance-trained | NR | randomized double-blind crossover | 5 mg/kg | 60 min pre-exercise | placebo | Controlled | 4 effects: Maximal strength (2); Muscular endurance (2) |
| **Wilk (2022)** | Poland | 12 women habituated to caffeine | resistance-trained | 23.3 | randomized crossover | 3 mg/kg; 6 mg/kg | pre-exercise | placebo | Not/unclear | 2 effects: Movement speed (2) |
| **Parks (2023)** | United States | 11 women | resistance-trained | 24 | double-blind placebo-controlled crossover | 4 mg/kg capped at 300 mg | 60 min pre-exercise | placebo | Not/unclear | 2 effects: Total load (2) |
| **Robles-González (2023)** | Spain | 15 resistance-trained women | resistance-trained | 25.1 | double-blind randomized crossover | 3 mg/kg | 60 min pre-exercise | placebo | Not/unclear | 10 effects: Maximal strength (4); Movement speed (4); Muscular endurance (2) |
| **Abumohd (2024)** | Jordan | 10 resistance-trained females | resistance-trained | 24.5 | randomized double-blind placebo-controlled crossover | 4 mg/kg | 60 min pre-exercise | placebo | Controlled | 4 effects: Muscular endurance (4) |
| **Prather (2024)** | United States | 36 resistance-trained women | resistance-trained | 22.3 | double-blind placebo-controlled crossover | 6 mg/kg | 30 min pre-exercise | placebo | Not/unclear | 8 effects: Maximal strength (4); Muscular endurance (4) |
| **Shlool-Abumohd (2024)** | Jordan | 11 strength-trained eumenorrheic females | strength-trained | 26.2 | randomized double-blind placebo-controlled crossover | 4 mg/kg | 60 min pre-exercise | placebo | Controlled | 2 effects: Muscular endurance (2) |
| **Snyder (2024)** | United States | 10 resistance-trained women | resistance-trained | 21.5 | randomized double-blind crossover | 6 mg/kg | pre-exercise | placebo; multi-ingredient pre-workout | Not/unclear | 4 effects: Muscular endurance (2); Total load (2) |
| **Kardas (2025)** | Poland | 13 female soccer players | trained team-sport athletes | 21.1 | randomized double-blind crossover | 6 mg/kg | pre-exercise | placebo | Not/unclear | 3 effects: Functional strength (3) |

**Abbreviations.** NR, not reported; mg·kg⁻¹, milligrams per kilogram of body mass; 1RM, one-repetition maximum.

**Supplementary document 3.**

## Supplementary Table S3. Complete database search strategies

## PubMed (141 records) (caffeine[MeSH Terms] OR caffeine[Title/Abstract] OR caffeinated[Title/Abstract] OR coffee[Title/Abstract] OR "energy drink*"[Title/Abstract] OR "pre workout"[Title/Abstract] OR preworkout[Title/Abstract]) AND (female[MeSH Terms] OR female*[Title/Abstract] OR women[Title/Abstract] OR woman[Title/Abstract] OR girl*[Title/Abstract] OR menstrual[Title/Abstract] OR menstruating[Title/Abstract] OR "menstrual cycle"[Title/Abstract] OR follicular[Title/Abstract] OR luteal[Title/Abstract] OR "oral contraceptive*"[Title/Abstract]) AND ("Resistance Training"[MeSH Terms] OR "Weight Lifting"[MeSH Terms] OR "Muscle Strength"[MeSH Terms] OR "resistance exercise*"[Title/Abstract] OR "resistance training"[Title/Abstract] OR "strength training"[Title/Abstract] OR "weight training"[Title/Abstract] OR weightlifting[Title/Abstract] OR "weight lifting"[Title/Abstract] OR "bench press"[Title/Abstract] OR squat[Title/Abstract] OR deadlift[Title/Abstract] OR "leg press"[Title/Abstract] OR "knee extension"[Title/Abstract] OR "elbow flexion"[Title/Abstract] OR "maximal strength"[Title/Abstract] OR "one repetition maximum"[Title/Abstract] OR 1RM[Title/Abstract] OR "repetitions to failure"[Title/Abstract] OR "muscular endurance"[Title/Abstract] OR "muscle power"[Title/Abstract] OR "movement velocity"[Title/Abstract] OR "bar velocity"[Title/Abstract]) AND (random*[Title/Abstract] OR placebo[Title/Abstract] OR trial[Title/Abstract] OR crossover[Title/Abstract] OR cross-over[Title/Abstract] OR "controlled trial"[Title/Abstract]) NOT (animals[MeSH Terms] NOT humans[MeSH Terms]).

## Web of Science Core Collection (153 records) TS=(caffeine OR caffeinated OR coffee OR "energy drink*" OR "pre workout" OR preworkout) AND TS=(female* OR women OR woman OR girl* OR menstrual OR menstruating OR "menstrual cycle" OR follicular OR luteal OR "oral contraceptive*") AND TS=("resistance exercise*" OR "resistance training" OR "strength training" OR "weight training" OR weightlifting OR "weight lifting" OR "bench press" OR squat OR deadlift OR "leg press" OR "knee extension" OR "elbow flexion" OR "maximal strength" OR "one repetition maximum" OR 1RM OR "repetitions to failure" OR "muscular endurance" OR "muscle power" OR "movement velocity" OR "bar velocity") AND TS=(random* OR placebo OR trial OR crossover OR "cross-over" OR "controlled trial").

## Scopus(63 records)

## TITLE-ABS-KEY(caffeine OR caffein*) AND TITLE-ABS-KEY(female* OR woman OR women OR girl*) AND TITLE-ABS-KEY("resistance exercise" OR "resistance training" OR "strength training" OR "weight training" OR "weight lifting" OR "strength exercise") AND TITLE-ABS-KEY(strength OR "one repetition maximum" OR "one-repetition maximum" OR 1RM OR "muscular endurance" OR endurance OR velocity OR "movement speed" OR "bar velocity" OR power OR repetitions OR "total load") AND TITLE-ABS-KEY(placebo OR random* OR crossover OR "cross-over" OR trial)

## Cochrane Library (62 records)

## #1 caffeine:ti,ab,kw OR caffein*:ti,ab,kw

## #2 female*:ti,ab,kw OR woman:ti,ab,kw OR women:ti,ab,kw OR girl*:ti,ab,kw

## #3 "resistance exercise":ti,ab,kw OR "resistance training":ti,ab,kw OR "strength training":ti,ab,kw OR "weight training":ti,ab,kw OR "weight lifting":ti,ab,kw OR weightlifting:ti,ab,kw OR "strength exercise":ti,ab,kw

## #4 strength:ti,ab,kw OR "muscle strength":ti,ab,kw OR "maximal strength":ti,ab,kw OR "maximum strength":ti,ab,kw OR "one repetition maximum":ti,ab,kw OR "one-repetition maximum":ti,ab,kw OR 1RM:ti,ab,kw OR "muscular endurance":ti,ab,kw OR "muscle endurance":ti,ab,kw OR endurance:ti,ab,kw OR velocity:ti,ab,kw OR "movement speed":ti,ab,kw OR "bar velocity":ti,ab,kw OR power:ti,ab,kw OR repetitions:ti,ab,kw OR reps:ti,ab,kw OR "total load":ti,ab,kw

## #5 placebo:ti,ab,kw OR random*:ti,ab,kw OR trial:ti,ab,kw OR crossover:ti,ab,kw OR "cross-over":ti,ab,kw OR "cross over":ti,ab,kw

## #6 #1 AND #2 AND #3 AND #4 AND #5、

## Supplementary Table S4. Domain-level GRADE judgments

| Outcome | Risk of bias | Inconsistency | Indirectness | Imprecision | Publication bias | Overall certainty |
| --- | --- | --- | --- | --- | --- | --- |
| Maximal strength | no included cluster was low overall risk; common concerns in randomization, carryover, and reporting. | Not further : average effect was comparatively stable, although PI crossed zero. | Not : direct female/extractable female resistance-exercise evidence. | small effect and PI compatible with no effect. | Not further , but Egger/funnel findings treated as exploratory. | Low |
| Muscular endurance | ommon RoB concerns across contributing crossover trials. | substantial heterogeneity and very wide PI. | Not : direct outcome domain, but protocols varied. | CI and PI compatible with smaller, null, or unfavorable future effects; interpretation remains cautious because reproducibility is uncertain. | Possible small-study effects; interpreted cautiously. | Low |
| Movement speed | RoB concerns and few independent clusters. | Serious concern: heterogeneity not reliably decomposed with only three clusters. | imited replication across protocols. | sparse clusters and uncertain CR2 inference. | Exploratory only because too few clusters. | Very low |
| Functional endurance | single cluster. | Not estimable. | single fixed-dose middle-aged sample. | sparse evidence. | Not assessable. | Very low |
| Functional strength | single cluster. | Not estimable. | single soccer-player trial. | sparse evidence. | Not assessable. | Very low |
| Total load | two clusters with RoB concerns. | Not reliably estimable. | limited protocol coverage. | sparse and imprecise evidence. | Not assessable. | Very low |
